# Understanding culture and HIV/AIDS in sub-Saharan Africa

**DOI:** 10.1080/17290376.2013.807071

**Published:** 2013-06-25

**Authors:** Steven Sovran

**Keywords:** culture, HIV/AIDS, sub-Saharan Africa, population risk, VIH, SIDA, profil épidémiologique, Afrique subsaharienne, épidémie, causalité culturelle, risque individuel, risque attribuable dans la population, hypothèse ethnocentrique, hypothèse biaisée

## Abstract

Early in the study of HIV/AIDS, culture was invoked to explain differences in the disease patterns between sub-Saharan Africa and Western countries. Unfortunately, in an attempt to explain the statistics, many of the presumed risk factors were impugned in the absence of evidence. Many cultural practices were stripped of their meanings, societal context and historical positioning and transformed into cofactors of disease. Other supposedly beneficial cultural traits were used to explain the absence of disease in certain populations, implicitly blaming victims in other groups. Despite years of study, assumptions about culture as a cofactor in the spread of HIV/AIDS have persisted, despite a lack of empirical evidence. In recent years, more and more ideas about cultural causality have been called into question, and often disproved by studies. Thus, in light of new evidence, a review of purported cultural causes of disease, enhanced by an understanding of the differences between individual and population risks, is both warranted and long overdue. The preponderance of evidence suggests that culture as a singular determinant in the African epidemic of HIV/AIDS falls flat when disabused of its biased and ethnocentric assumptions.

## Abstract (FR)

Aux debuts des etudes sur le VIH/SIDA, la culture a été invoquée pour expliquer les différences entre le profil épidémiologique de lAfrique subsaharienne et celui des pays occidentaux. Malheureusement, dans une tentative d'expliquer les statistiques, bon nombre des facteurs de risque présumés ont été contestés en l'absence de preuve. De nombreuses pratiques culturelles ont été dénuées de leur sens, et de contexte social et historique pour être transformées en cofacteurs de la maladie. D'autres traits culturels censément bénéfiques ont servi à expliquer l'absence de maladie au sein de certaines populations, ce qui, de manière implicite, a lancé le blâme sur les victimes d'autres groupes. Malgré des années d'études et le manque de preuve empirique, les présomptions selon lesquelles la culture serait un cofacteur dans la propagation du VIH/SIDA persistent. Au cours des années récentes, de plus en plus de théories sur la causalité culturelle ont été remises en question et souvent, ont été réfutées par des études. Ainsi, à la lumière de nouvelles preuves, un examen des prétendues causes culturelles de la maladie, reposant sur une compréhension des différences entre le risque individuel et le risque attribuable dans la population est non seulement justifié, mais également attendu depuis fort longtemps. Selon la prépondérance des preuves, considérer la culture comme seul déterminant de l'épidémie de VIH/SIDA en Afrique ne tient pas quand les hypothèses biaisées et ethnocentriques sont écartées.

The onslaught of HIV/AIDS in sub-Saharan Africa provides an unparalleled opportunity to examine the complex relationship between culture and disease (Airhihenbuwa & Webster 2004). Culture was invoked early in the epidemic in sub-Saharan Africa to explain the spread of HIV in the heterosexual population at a time when infection was predominantly spread in the developed world amongst injection drug users and homosexual men (Hrdy 1987; Packard & Epstein 1991). The sex parity in African infection rates, which stood in stark contrast to the male preponderance of infection in the USA and Europe (Gausset 2001; Hrdy 1987; Hunt 1996; Manners 1985), also drew researchers' attention. Analysts soon turned to culture as a means of accounting for these dissimilarities, often ignoring the powerful effects of structural factors (such as poverty). Given the limited knowledge base, culture in sub-Saharan Africa, considered exotic or ‘primitive’, was often inappropriately vilified based on erroneous and prejudicial assumptions (Gausset 2001; Packard & Epstein 1991). These assumptions, previously taken for granted, are increasingly being undermined by new findings and reconceived as having little effect on HIV rates. A renewed look at the supposed cultural cofactors of HIV/AIDS, in light of years of research, is therefore warranted in order to elucidate their true significance.

## Examining culture and HIV/AIDS

Culture and HIV/AIDS in sub-Saharan Africa are linked by a tangled web of associations that are difficult, if not impossible, to isolate for the purposes of study. Any such analysis will suffer from important shortcomings, at the root of which lies the notion of culture itself, a contested and problematic concept plagued by difficulties pertaining to both definition and application. This paper neither attempts to put to rest the decades-long debates about the nature of culture (Brightman 1995), nor proposes its own unifying definition of culture, but rather relies on the authors in the literature to determine which factors associated with the HIV/AIDS pandemic are presumed to be cultural in nature. Even though the purportedly cultural factors herein presented are debatably ‘cultural’ in nature, they have all been presented as such in the literature to varying degrees by the authors. Their treatment as cultural by many authors at a minimum suggests that they are often understood, treated and addressed as cultural factors, often by people intimately involved in the struggle against HIV/AIDS. Once purported ‘cultural cofactors of disease’ were identified in the literature, an extensive search was undertaken (using appropriate keywords in databases of social science, natural science and humanities literature available at a major research university) to examine the body of evidence gathered that either supported or refuted the presumed role of each cultural trait or phenomenon. This iterative process often led to the identification of further cultural factors and the process was repeated. The summary of the evidence pertaining to each topic is provided in turn below, including studies conducted in sub-Saharan Africa since the first description of HIV/AIDS but with a focus on the most recent. The cultural factors considered include traditional practices involving blood and other body fluids, sexual norms, early marriage and coital debut, widow inheritance and sexual cleansing rituals, gender relations and norms, female genital cutting (FGC), male circumcision and religion or religiosity.

Any attempt to examine culture in a manner akin to an independent variable in the spread of HIV may be challenged as invalid and indefensible in social analysis. These criticisms are certainly not without merit. A review of this nature almost inevitably implies that potential cultural determinants of infection are perfectly measurable, which they are often not. Furthermore, explorations into links between culture and any disease are inherently reductionistic, oversimplify the complex nature of cultural phenomena, essentialise groups of individual agents, misrepresent culture as static and falsely imply cultural homogeneity in the entirety of a region. The list of valid reservations undoubtedly goes on. The critics, however, often ignore the fact that elements presumed to be cultural (and more specifically ‘traditional’) are actively being considered in global health circles as loci for intervention (AIDSnet & Bruun 2006). Thus, an examination of the evidence behind the proposed link between a cultural trait and HIV is a practical and useful exercise that can rely on the best available evidence, imperfect though it may be. In addition, the preponderance of evidence herein discussed in fact supports a de-emphasis of the importance of cultural drivers of the pandemic, in the end supporting the academic criticisms. For these reasons, a compilation of existing information on the causal contributions of specific cultural practices that have been implicated in the spread of HIV in sub-Saharan Africa is not only desirable but long overdue.

This review benefits from an understanding of individual and population risks of disease. The distinction between the two, as proposed in a groundbreaking paper by Rose (1985), is fundamental to the consideration of the causation of any pathological process. Individual risk refers to the chances, given an individual's exposure to a causative agent, of developing the disease in question. It is not always directly related to population risk, which depends on the aggregate exposure to a cause in an entire population. Individual risk factors can be identified by comparing the prevalence of disease in exposed and unexposed subgroups of individuals. Population risk factors, however, can only be recognised through the same comparison at the level of population aggregates. Thus, a behaviour that makes an individual vulnerable to HIV infection does not always translate into population statistics. Individual risk factors must be either sufficiently potent or sufficiently common, or both, in order to have a discernible effect on the population as a whole. Population risk factors, on the other hand, must show sufficient heterogeneity in individual exposure rates within the population in order to translate into differences in individual risk. (This is because, at the extreme, if everyone in a population is exposed to the same degree, then no one individual is more vulnerable than any other.) A proposed population risk factor must also differ in prevalence between populations; otherwise, it cannot be used to explain a discrepancy in disease burden between these same populations. Individual and population risks have frequently been conflated in discussions regarding culture and HIV/AIDS, thereby obscuring reality since the reasons that an individual has acquired HIV cannot necessarily be invoked to explain why sub-Saharan Africa as a whole has high rates of HIV.

## Culture viewed as a determinant of HIV prevalence

### Practices involving blood or other body fluids

Traditional surgical practices, such as scarification, male circumcision and genital tattooing, are identified in the literature as potential sources of infection, especially if performed on groups (Feldman 1990; Hrdy 1987) and if the serial use of unsterilised equipment features prominently, as was the case in a rural Nigerian population studied by Ajuwon, Brieger, Oladepo and Adeniyi (1995). In Ajuwon *et al.*'s research setting, an indigenous medical practitioner known as an *olólà* performed male circumcision, tattooing and facial scarification after simply rinsing his knife in water between patients. This practice quite possibly exposes subsequent patients to the bodily fluids of previous patients. One must also consider the possibility of traditional healers being themselves infected with HIV, implying that they may act as a source of infection if they have open wounds.

The conclusions of Ajuwon *et al.* (1995) should of course not be construed as representative of all sub-Saharan Africa, but in the absence of epidemiological data, a close inspection of the case study does serve to problematise assumptions about the potency of traditional surgical practices as vectors of transmission. For instance, there were only a small number of clients who underwent operations or incisions sequentially. There was also a significant time lapse between operations, making transmission via unsterilised equipment unlikely because of the general instability of the virus in the extracorporeal environment (Ajuwon *et al.* 1995; Packard & Epstein 1991). Moreover, almost all of the *olólà*'s procedures were performed on infants and children, who have a lower prevalence of HIV. Based on experience in Zambia and Tanzania, Gausset (2001) believes that razor blades are available and affordable and that most people bring their own blade when visiting a healer. Altogether, traditional medical practices are thought to be relatively weak risk factors for HIV transmission and engender a relatively low individual risk to each patient. The low potency of these practices to transmit HIV suggests that they also have only minor effects on population risk and, therefore, do not account for the disparate HIV burden in sub-Saharan Africa.

### Sexual norms

Any sexually transmitted disease inevitably raises questions about sexual practices. The perceived sexual promiscuity of people in Africa, as compared to those in the West, has been frequently blamed for the rapid and extensive spread of the virus (Gausset 2001; Hunt 1996). Africans are often believed to be more culturally tolerant of sexual indulgence, multiple sexual partnerships and prostitution than people in other parts of the world (Hrdy 1987; Packard & Epstein 1991). Caldwell, Caldwell and Quiggin (1989) posited that Africans did not see sexual behaviour as a moral issue and thus had patterns of sexual behaviour that differed markedly from the West. Sexual initiation rites that promote liberal approaches to sexuality were also blamed in part for creating a permissive environment for promiscuity and for directly providing opportunities for HIV transmission. Nkwi (2005) point to culturally sanctioned indiscriminate sexual behaviour at rituals in Kenya, while Macdonald (1996) suggests that attitudes supportive of fertility encourage multiple partnerships and unsafe sex in Botswana.

Packard and Epstein (1991) call this preoccupation with promiscuity in exotic groups a fixation on the ‘sexual life of the natives’. They posit that the early fixation on African promiscuity and sexual deviance stemmed from the observation that in the USA and Europe, HIV was spreading in homosexual communities, which were widely assumed to be promiscuous (Packard & Epstein 1991). The African situation was seen as analogous. Since exposure to greater numbers of sexual partners over a lifetime constitutes an obvious source of vulnerability to HIV, this theory was not illogical, but teeming with assumptions and ultimately incorrect. As Halperin and Epstein (2004:4) note, studies have shown that Africans ‘report roughly similar, if not fewer, numbers of lifetime partners than do heterosexuals in many western countries’. These findings, however, did not exonerate the ‘culture of sex’ in Africa, and the impulse to find the root of HIV/AIDS in African sexual practices continued.

Attention turned to concurrency in sexual relations, including extramarital affairs, multiple concurrent partnerships and polygamy. Though the number of lifetime sexual partnerships may be on par with other parts of the world, researchers proposed that Africans differ in their acceptance of multiple concurrent sexual partnerships (Halperin & Epstein 2004, 2007; Morris & Kretzschmar 1997; Nyindo 2005). Logically, the interconnection of several sexual relationships via partnership concurrency can spread HIV to many more individuals more rapidly. In fact, mathematical modelling has demonstrated that having multiple partners concurrently increases the propagation of HIV through a population exponentially (Johnson, Dorrington, Bradshaw, Pillay-Van Wyk & Rehle 2009; Morris & Kretzschmar 1997), though the validity of the mathematical model has been questioned by Sawers and Stillwaggon (2010).

There are several problems with these accusations. For example, as argued by Gausset (2001), polygamy alone does not spread HIV since a polygamous family in which all partners are faithful is no more threatening than a monogamous marriage in the same circumstances. In fact, according to Sawers and Stillwaggon (2010), at least four ecological studies have shown no statistical connection between partner concurrency and HIV (Lagarde, Enel, Seck, Gueye-Ndiaye, Piau, Pison, *et al.* 2000; Mishra & Bignami-Van Assche 2009; Reniers & Watkins 2010; Tanser, Barnighausen, McGrath, Garnett & Newell 2009). The available evidence does not support the idea that partner concurrency inevitably imparts greater risk of HIV. Furthermore, none of the speculation proves that people in sub-Saharan Africa engage in multiple concurrent partnerships more frequently than elsewhere, which would be requisite if it were to explain population differences in HIV prevalence. In 2006, a study of sexual behaviour from a global perspective directly challenged these assumptions by revealing that rates of concurrency were lower in Africa than in many developed countries (Wellings, Collumbien, Slaymaker, Singh, Hodges, Patel, *et al.* 2006). In their thoroughly researched systematic review of the topic, Sawers and Stillwaggon (2010) conclude that partner concurrency has neither been shown to increase the epidemic spread of HIV more than other forms of sexual organisation, nor has it been shown to be more common in the African region than elsewhere.

Thus, theories regarding both promiscuity and concurrency of partnerships amongst Africans have proved untrue. This does not deny the important role of sexual practices in spreading HIV, especially insofar as greater sexual exposure imparts greater individual risk. Heterosexual contact, after all, remains the primary mode of transmission for HIV in sub-Saharan Africa. Rather, promiscuity and concurrent partnerships cannot serve as explanations for the population burden of HIV in sub-Saharan Africa as compared to other world regions when many developed countries have similar or even higher rates of these behaviours. Risk factors, as noted, can only be used to explain differences in population risk if they differ systematically between populations with low prevalence and those with a high prevalence of disease. Though the same level of sexual exposure carries different risks in regions with high and low ambient prevalence of HIV, it cannot be used to explain why prevalence statistics came to be so disparate in the first place. Therefore, though sexual exposure remains the primary conduit for the virus, other factors must act to potentiate its spread through sexual networks.

### Early marriage and coital debut

According to some authors, culture helps determine not only which sexual relations are acceptable, but also at what times and under what circumstances (Hrdy 1987; Nkwi 2005). An early onset of sexual activity, whether within or without marriage, was noted in the literature to imperil the individual by extending the period of potential exposure to sexually transmitted pathogens, while the immaturity of reproductive organs may further enhance an individual's biological susceptibility (Ghebremichael, Larsen & Paintsil 2009; Hallett, Lewis, Lopman, Nyamukapa, Mushati, Wambe, *et al.* 2007). Younger women, on average, may also be less likely to insist on safe sexual practices, such as the use of a condom, when negotiating sexual encounters, particularly with older men (Hallett, Lewis, *et al.* 2007; Pettifor, van der Straten, Dunbar, Shiboski & Padian 2004). A World Health Organization (WHO) study found that females under the age of 19 were more likely than older women to experience forced first sex as well as physical and sexual violence at the hands of a partner (UNAIDS 2010).

In a 2004 study of women from Zimbabwe, Pettifor and her co-investigators (2004) reported that an age at first sex under 15 correlated with higher lifetime numbers of sexual partners, a lack of high school education and engagement in transactional sex work. These same women, however, were more likely to have ever used a condom. Overall, the women with early debut were more likely to be HIV positive at a rate of 54.6% compared to 38.2% for those with a later debut. In a separate study, Hallett, Lewis, *et al.* (2007) found that an early coital debut in Zimbabwean women, if outside the context of marriage, conferred a greater vulnerability to HIV. A third study of almost 2000 women in Northern Tanzania confirmed a statistically significant association between early coital debut and HIV positivity (Ghebremichael *et al.* 2009). Furthermore, reports of forced first sex, 55% of which occurred before age 18, were common among the Tanzanian women and were correlated with subsequent high-risk sexual behaviours. Finally, an examination of almost 12,000 youths aged 15–24 from South Africa published in 2009 confirmed the links between early sexual debut and having an older partner, experiencing forced or coerced intercourse and omitting condoms at first intercourse (Pettifor, O'Brien, MacPhail, Miller & Rees 2009). Though measured rates of HIV positivity were not included in this study's protocol, these experiences are known to increase vulnerability to HIV.

Even if early sexual debut occurs within marriage, young women are not spared the enhanced vulnerability of acquiring HIV. A multicentre study in Kenya and Zambia has found that married adolescent girls had higher rates of HIV prevalence than unmarried, sexually active girls in the same age cohort (Clark 2004). Though the finding may initially seem surprising, given assumptions about the safety of sexuality within marriage, the phenomenon may be explained by decreased condom use, increased frequency of sexual contact and impairment of a woman's ability to refuse sex within marriage. Marriage also tends to pair older men, a demographic with higher rates of HIV, with younger women. These results do not challenge the importance of faithfulness within marriage, but rather discredit assumptions that marriage is automatically protective.

These studies have clearly shown that vulnerability to HIV and thus individual-level risk increase with early coital debut. Whether the increased risk associated with early debut can explain discrepancies in HIV population-level prevalence on a global scale is, however, another consideration. One must take into account evidence indicating that the age at first intercourse in sub-Saharan Africa does not differ markedly in comparison to other parts of the world that have not suffered from HIV to the same degree (Wellings *et al.* 2006). Even though they confer vulnerability, rates of early marriage or coital debut do not vary between populations that do demonstrate variation in HIV positivity. On a population level, therefore, these behaviours cannot be used to explain the higher rates of HIV in sub-Saharan Africa. In addition, mathematical modelling has estimated that, in fact, the benefit of delay in sexual initiation on a population level is relatively small (Hallett, Gregson, Lewis, Lopman & Garnett 2007). Therefore, the assumption that sub-Saharan Africans initiate sex at an earlier age for cultural reasons and that this phenomenon helps to explain the high prevalence of HIV/AIDS in the region once again does not stand up to the evidence.

### Widow inheritance and sexual cleansing rituals

In some groups, widow inheritance, or levirate marriage, usually by a brother of the deceased husband, helps to maintain the social and economic welfare of widows and orphans (Barnett & Parkhurst 2005; Mabumba, Mugyenyi, Batwala, Mulogo, Mirembe, Khan, *et al.* 2007). The family of the deceased may also desire continued control over the widow and the dowry, as well as any wealth accumulated by the deceased (Mabumba *et al.* 2007). Widow inheritance has been implicated in the spread of HIV/AIDS because it encourages the formation of extended sexual networks (Nyindo 2005). The considerable chance that the death of a young husband was caused by AIDS means that widows may also be more likely to be infected (Ayikukwei, Ngare, Sidle, Ayuku, Baliddawa & Greene 2008; Nyindo 2005). Over two-thirds of respondents in a rural Ugandan study reported the existence of widow inheritance in their communities, though less than a third supported the practice (Mabumba *et al.* 2007). Lugalla, Emmelin, Mutembei, Sima, Kwesigabo, Killewo, *et al.* (2004) in fact found that widow inheritance was becoming obsolete in the Kagera region of Tanzania. Unfortunately, no studies have directly assessed the effect of widow inheritance on the likelihood of HIV positivity.

In other settings, sexual contact with a widow is encouraged through sexual cleansing rituals, in which penetrative intercourse is thought to chase away the spirit of the deceased and thereby prevent misfortune amongst the living (Malungo 2001; Nkwi 2005). Widows who are tainted by death are ritually accepted back into the community and relieved of evil (Ayikukwei, Ngare, Sidle, Ayuku, Baliddawa & Greene 2007). In some cases, for example, among the Luo of Kenya, a hired cleanser may be paid to perform the ritual (Ayikukwei *et al.* 2007, 2008). These cleansers engage in unprotected sex with multiple partners, possibly in the same night if cleansing the widows of a polygamous man (Ayikukwei *et al.* 2007). In the case where a wife dies, her widower is sometimes considered unclean. In order to be cleaned, he must first dream of having sexual intercourse with his dead wife and then find a new woman with whom to have sex (Ayikukwei *et al.* 2007).

Despite the persistence of these rituals and beliefs, Malungo (2001) found that alternative practices to sexual cleansing were becoming common in Zambia, particularly amongst younger age groups. These alternative rituals include sliding over a partially naked person, administrating herbs, hair-cutting and offering prayers. Malungo attributes the changing practices to the advent of HIV/AIDS, as well as to the influence of Western religion, education and modernity. Ayikukwei and her colleagues (2007) also note the increasingly common practice of symbolic cleansing amongst the Luo, in which a cleanser spends the night with the widow but does not perform sexual intercourse. Similarly, new practices are replacing widow inheritance. For example, in Burkina Faso, some inheritors will publicly proclaim a marriage to a widow, but if she tests positive for HIV, the inheritor will not engage in sexual intercourse with her, while in Uganda, Christian values of monogamy are being used to curb the practice of widow inheritance (AIDSnet & Bruun 2006).

Since HIV is potentially transmitted after widow inheritance or during sexual cleansing rituals, the practices pose a risk to those individuals involved. Given the present state of knowledge, however, the population-level risk associated with widow inheritance and sexual cleansing is difficult to determine. There are no reliable data to indicate how common or how dangerous the practices are. In particular, prevalence statistics in large national or subnational population aggregates are absent. The nature of sex may also be an important determinant of vulnerability, since one-time sexual contact is less dangerous than ongoing encounters. Furthermore, the levirate may not increase risk if widows would have remarried or engaged in sex with other partners anyway. Thus, key information is lacking, leaving the association between HIV and widow inheritance or cleansing obscure. Claims that these cultural practices explain differences in HIV prevalence are thus unsubstantiated and speculative at best.

### Gender relations and norms

Culturally sanctioned gender relations have an especially prominent role in the HIV/AIDS epidemic in sub-Saharan Africa, where HIV rates in women substantially exceed those in men (UNAIDS 2008). Generally, in the literature, gender is understood as the social role occupied by each sex and gender relations as the interactions between these two social roles. The relative status of women in society in general and in their intimate relationships in particular can strongly impact the chances of being infected (Macdonald 1996) and is a common theme in the literature. In fact, gender inequality has been accused of being the primary factor that determines patterns of HIV in Africa (Niëns & Lowery 2009). If women find themselves in a subordinate position to male sexual partners, particularly with the threat of violence, they may be unable to refuse unsafe sexual practices, to insist on condom usage, to resist rape and to control their male partners' faithfulness (Jenkins 2004; Macdonald 1996; Niëns & Lowery 2009). The low status of women has also been connected to poor participation in HIV education and prevention programmes (Duffy 2005). In some cases, pronatalist beliefs may pressure women into proving their fertility by becoming pregnant before marriage, thus promoting pre-marital unsafe sex (Macdonald 1996). Women in sub-Saharan Africa also often have older male partners who are more likely to be HIV positive (UNAIDS 2010). Furthermore, when the dissolution of an unhealthy relationship is not a culturally viable option for women, their vulnerability to HIV infection is compounded.

Niëns and Lowery (2009) tested the hypothesis that gender inequality and HIV prevalence are positively correlated using the Gender-Related Development Index (GDI) compiled by the United Nations Development Programme. The study first compared gender equality by country with the absolute national HIV prevalence. The GDI was found to correlate negatively with point prevalence of HIV, meaning that gender equality decreases HIV rates on a population level. Changes in HIV prevalence between 2000 and 2005 were then compared to changes in GDI over the same period. The researchers found that as gender equality increased, the prevalence of HIV fell. The strength of the association only increased when the data were controlled for economic development, education levels, quality of the health care system and religion. Thus, the study suggests not only that gender inequality and HIV are strongly linked, but also that improvements in gender relations can have a positive impact in stemming the spread of HIV in a population.

Ethnographic evidence in relatively egalitarian societies also supports the connection between gender relations and HIV. Amongst the Ju/'hoansi in Namibia and Botswana, who are conspicuous both for relative gender equality and for a comparatively low prevalence of HIV/AIDS, the autonomy of women has been credited with containing the spread of HIV (Lee 2007; Susser 2009). In 2001, estimates set the prevalence of HIV at 3.3% of Ju/'hoan men and women aged 15–49, while the same age group had national prevalence of 38% and 22.5% in the general populations of Botswana and Namibia, respectively (Lee 2007). Beginning in the 1960s, extensive ethnographic fieldwork of the traditionally nomadic hunter-gatherer group has documented the high level of female autonomy (Lee 2007; Susser 2009). Women were empowered to veto marriage plans, to divorce their husbands and even to participated in tribal councils or decision-making. In addition, women's role as gatherers granted them significant economic clout since they provided up to 70% of the food for the group (Lee 2007). Despite the challenges posed by the arrival of missionaries, the modern state and capitalist interests, Ju/'hoan women still maintain significant authority and self-determination to this day, especially with respect to sexuality (Susser 2009). They generally express a greater confidence in relations with men as compared to surrounding ethnic groups (Susser 2009). Women, for example, are generally entitled to insist on the use of a condom or to refuse sex. The gender relations amongst the Ju/'hoansi can, therefore, credibly be invoked to help explain their low prevalence of HIV positivity.

Mane and Aggleton (2001) put forth a complementary argument regarding the dangers of gender inequality experienced by men owing to cultural norms of behaviour. They point out that ‘hegemonic forms of masculinity oppress not only women but also men – constraining what they can and cannot think and do’ (2001:29). Men's identities and behaviours are also undoubtedly shaped by culture. For example, men consistently have higher rates of changing sexual partners than women, a behavioural pattern that creates vulnerability to HIV. Expectations of ‘masculine’ sexual behaviour are often reinforced beginning in childhood and adolescence. Similarly, boys are often taught that ‘real’ men already know about sex (and see no need to inform themselves about safe sex), do not seek medical attention and use alcohol or drugs frequently (which can induce hazardous behaviours). Furthermore, homosexuality, which does not fit the definition of masculinity in many cultures, can be forced underground where treatment and prevention do not extend and where perilous behaviours are condoned in secret. These factors may explain why a meta-analysis of 23 cohort studies examining death rates by gender in patients enrolled in antiretroviral treatment programmes in Africa has concluded that men participate in such programmes less than women and that they experience an increased risk of death compared to women (Druyts, Dybul, Kanters, Nachega, Birungi, Ford, *et al.* 2013). The socioculturally mandated masculine identities result in very real vulnerabilities to HIV and may impair the success of antiretroviral treatment. The key, therefore, to mitigating gender-based vulnerability lies not in blaming men, but in changing the cultural and structural norms that define both femininity and masculinity.

### Female genital cutting (FGC)

FGC is often proposed in the literature to be predisposed to HIV infection through a variety of theoretical mechanisms. The surgery is traditionally performed in various ways, ranging from removal of the clitoral prepuce to radical excision of the vulva and subsequent partial closure of the vaginal introitus (Hrdy 1987). HIV could in theory be transmitted directly when performed using septic tools (Brady 1999; Hrdy 1987; Yount & Abraham 2007) or through the transfer of blood from the practitioner into the open wounds of the female (Brewer, Potterat, Roberts & Brody 2007). A Tanzanian study reported that the same unsterilised equipment was used to perform FGC on 15–20 girls sequentially, up to 97% of the time (Mutembei & Mwasiga 1998). Though the procedure tends to be performed on young women and girls who are less likely to have had their sexual debut and are thus also less likely to be HIV positive (barring treated cases of vertical transmission), anecdotal evidence of this mode of transmission exists (Brady 1999). After the procedure, the altered anatomy is often thought to enhance exposure to the virus during sexual intercourse. The scar tissue and abnormal anatomy resulting from FGC could predispose to vaginal trauma, which in turn compromises the protective functions of the vaginal lining for women (Hrdy 1987; Yount & Abraham 2007) and increases exposure to the woman's blood, and thus the virus, for men. In the most extreme forms of FGC, the vaginal introitus must be opened by incision for sexual intercourse or childbirth, which creates further scarring and may further increase susceptibility to infection. FGC can lead to mechanical difficulties or severe pain during intercourse, which in turn, as Linke (1986) and Brady (1999) suggest, promotes anal sex as an alternative to penile-vaginal penetration. Since the anal mucosa is biologically more susceptible to infection than the vaginal lining, anal sex tends to increase the likelihood of HIV transmission (Salim & Gita 1998). Thus, the practice of FGC has been proposed in the literature to propagate HIV through a variety of theoretical mechanisms.

The reality of this theoretical link between HIV/AIDS and FGC has been empirically tested in a number of studies. Klouman, Manongi and Klepp (2005) studied a sample of Tanzanian women of all ages, half of whom consented to gynaecological examination, allowing direct visualisation of FGC. After controlling for a decline in FGC rates in younger women, no statistical correlation was found between FGC and HIV. Brewer *et al.* (2007) explored the potential connection in self-reported virgins in Kenya, seeking to remove the confounding factor of sexual activity in order to examine direct consequences of the procedure of FGC. On examination of the data, the weak correlation was not statistically significant. Furthermore, the group found that sexually active older females with FGC were in fact less likely to be infected with HIV than uncut women. Similarly, Yount and Abraham (2007) concluded that cut women were less likely to have HIV than uncut women in a Kenyan study. A further adjustment for social and demographic factors revealed parity in the two groups' infection rates. In 2009, Maslovskaya, Brown and Padmadas (2009) used the Kenyan Health and Demographic Survey, which included 3114 women, to find that women who underwent FGC had a lower prevalence of HIV than those who did not. The only correlation the researchers uncovered in subgroup analysis connected HIV/AIDS with first-union partner age. As compared to uncut women in the same situation, cut women with an older first partner were less likely to have HIV, while those with a same-age or younger first partner were more likely to have HIV. All results considered, the authors conclude that any association between FGC and HIV should not be interpreted as causative: a conclusion equally applicable to all these studies.

Though the theoretical plausibility of FGC posing an individual-level risk remains, the evidence challenges the importance of FGC as a major population-level driver of the HIV/AIDS in sub-Saharan Africa. If an association between HIV and FGC exists at all, it is likely to be unquantifiably weak, since the threat posed to an individual woman is not strong enough to be discernible in statistical data. FGC, then, is best interpreted as a theoretical individual risk factor, not supported by evidence. Furthermore, it certainly does not translate into a population risk for the purposes of understanding global disparities in HIV/AIDS prevalence. Though the practice may be impugned on other grounds, claims that FGC confers vulnerability to HIV transmission, according to the evidence, likely reflect cultural biases rather than biological fact.

### Male circumcision

Male circumcision has been identified as a protective cultural factor (Auvert, Taljaard, Lagarde, Sobngwi-Tambekou, Sitta, Puren, *et al.* 2005; Bailey, Moses, Parker, Agot, Maclean, Krieger, *et al.* 2007; Bongaarts, Reining, Way & Conant 1989; Gray, Kigozi, Serwadda, Makumbi, Watya, Nalugoda, *et al.* 2007; Halperin & Bailey 1999; Moses 2009; Siegfried, Muller, Deeks & Volmink 2009; Weiss, Quigley & Hayes 2000). Far from being a recently introduced phenomenon, over 400 indigenous ethnic groups traditionally practise some form of male circumcision in Africa, particularly in West African and Muslim countries (Bongaarts *et al.* 1989; Weiss *et al.* 2000). In fact, some West African tribes have been performing ritual circumcision for over 5000 years (Warner & Strashin 1981). Early observations of relative vulnerabilities suggested that circumcision might provide some degree of protection against the virus (Moses 2009). Based on these observations, differences in circumcision status were invoked to explain the discrepancies in HIV prevalence in separate but contiguous ethnic groups who varied in circumcision status but were exposed to roughly similar factors otherwise (Halperin & Bailey 1999).

These suggestions were soon corroborated using statistical correlations between lower population prevalence of circumcision and a higher prevalence of HIV (Bongaarts *et al.* 1989). In 2000, Weiss and her colleagues (2000) performed a meta-analysis of 28 studies on the topic. The group concluded that circumcision was highly statistically associated with a reduced vulnerability to HIV, especially for high-risk groups. However, the studies in the meta-analysis, given their observational nature, were unable to control for confounding factors, such as sexual behaviour patterns (Moses 2009; Weiss *et al.* 2000).

Thus, in order to clarify the issue, three prospective randomised controlled trials were undertaken in South Africa, Uganda and Kenya to analyse the effect of male circumcision for the prevention of HIV infection through heterosexual contact (Auvert *et al.* 2005; Bailey *et al.* 2007; Gray *et al.* 2007; Siegfried *et al.* 2009). All three trials were terminated early due to statistical findings that warranted offering circumcision to all participants because of its preventative potency. Meta-analysis of these three trials suggests that circumcision can reduce HIV incidence by as much as 38–66% over 24 months with a low complication rate (Siegfried *et al.* 2009). On the other hand, a Ugandan randomised controlled trial of HIV-positive men demonstrates that the benefit seems not to extend to the female sexual partners of HIV-positive circumcised men, who did not show lower rates of infection than the partners of men in the control group (Wawer, Makumbi, Kigozi, Serwadda, Watya, Nalugoda, *et al.* 2009).

The preponderance of available evidence suggests that male circumcision is protective against HIV acquisition in men. Though the exact interaction remains obscure, the foreskin is thought to facilitate transmission because it has a higher density of target cells for the virus, is more prone to trauma during sexual intercourse and is more susceptible to other communicable diseases that are known to act as co-factors for HIV infection, including syphilis and herpes (Halperin & Bailey 1999). In the long run, women may also benefit from male circumcision if the practice lowers the ambient prevalence of HIV and consequently decreases the statistical chances of contact with the virus (Baeten, Celum, Coates, Baeten, Celum & Coates 2009). Altogether, some estimates suggest that 7.7 million infections and 3 million deaths in sub-Saharan Africa could be prevented through routine circumcision of males (Moses 2009; Williams, Lloyd-Smith, Gouws, Hankins, Getz, Hargrove, *et al.* 2006).

Circumcision, importantly, does not prevent HIV transmission reliably on the individual level, but rather in population statistics. Thus, individual risk remains a very real concern for circumcised men, who could easily negate the benefits of circumcision by increasing their exposure through unprotected sexual intercourse with greater numbers of partners. In her study of a Kenyan population, Mattson (2007) did not find an increase in such behaviours amongst circumcised men as a whole, though the individual who is under the impression that he has achieved surgical immunity from HIV is patently mistaken. Nonetheless, as a consequence of the potential benefits on a population level, the WHO now advocates routine circumcision as one among many tools for the prevention of HIV acquisition, thereby transforming a cultural practice into a preventative public health strategy (Moses 2009). Still, circumcision status cannot be invoked as *the* cause of disparities in global HIV prevalence when large populations that generally do not practise circumcision, as in Europe and Latin America, have a relatively low prevalence of HIV. On a global basis, therefore, HIV prevalence does not appear to vary systematically on a population level with prevalence of male circumcision, even though circumcision may reduce transmission in high-prevalence countries. Circumcision alone does not explain differences in HIV prevalence among different populations.

### Religion and religiosity

Sub-Saharan Africa embraces a rich diversity of indigenous and imported religious traditions. Since moral behavioural proscriptions often trace their sources to religious teachings, religion and a strong adherence to religious principles have been thought to protect against HIV/AIDS transmission (Agha, Hutchinson & Kusanthan 2006; Gray 2004; Lagarde *et al.* 2000; Takyi 2003; Trinitapoli & Regnerus 2006). Identification with religious organisations and traditions that promote abstinence or monogamy, for example, could conceivably decrease the likelihood of participation in vulnerability-enhancing behaviours. Alternatively, religious teachings may be detrimental by banning certain protective measures, such as condom use, or denying young people education about safe sex (Trinitapoli 2009). Supporters of religion as a protective factor also point out that religious affiliation fosters the creation of interpersonal networks that may enhance the diffusion of HIV-related information, while detractors think religion may in fact hinder the spread of information (Takyi 2003). Of course, the degree to which religious teachings are thought to be protective depends not only on the balance between helpful and harmful doctrines, but also on the degree to which religion is incorporated into daily life by adherents. Since religion has undeniably been a very potent motivator of behaviour and behavioural change throughout history, it may logically play an important role in determining patterns of HIV/AIDS.

Contrary to many assumptions, however, the results of available investigations have tended to downplay the linkage of HIV to religion and religiosity. Lagarde and his colleagues (2000) concluded from a study of Muslims, Christians and Animists in a rural community of Senegal that there was little difference in HIV-related knowledge between groups that consider religion important and those that do not. Furthermore, the data suggest that religiosity was negatively correlated with preventative behaviours. Similarly, Takyi (2003), reporting on a Ghanaian population, concluded that Christian women had greater knowledge about behaviours affecting HIV transmission than other religious groups but found no consistent correlation between religious affiliation and actual preventative behavioural change. Zambian data analysed by Agha *et al.* (2006) revealed an uncertain effect of religion: belonging to a conservative religious group may support some hazardous behaviours while lowering others. For example, members of conservative religious sects in Zambia tended to delay sexual initiation, but were also less likely to use a condom at first intercourse. Though Trinitapoli and Regnerus (2006) were somewhat more supportive of the protective role of religiosity in rural Malawi, reporting that married male participants in religious services were less likely to report a recent extramarital partner, a sexually transmitted infection or a perceived high likelihood of contracting HIV in the future, the HIV status of participants was not determined in the course of the study. High-risk behaviour is likely to be underestimated by self-reporting and perceptions of risk. Later, Trinitapoli (2009) found that religion in Malawi had a negligible role in the promotion of abstinence, faithfulness and condom use, despite the efforts of the clergy to promote some or all of these behaviours. Muula (2010) similarly found no relationship between religious denomination or church attendance and HIV status or condom use in a study of Christian women in Malawi. Subsequently, Muula, Thomas, Pettifor, Strauss, Suchindran and Meshnick (2012), using a database of 2609 women in Malawi, failed to find any differences in HIV prevalence based on religious affiliation (either between Christians or Muslims or amongst various Christian denominations). Though many of these studies rely on metrics that do not necessarily correlate to absolute prevalence of HIV (such as knowledge of HIV, self-reports of behavioural change and decreased subjective perceptions of vulnerability), the preponderance of evidence still points to a negligible effect of religion and religiosity on the spread of HIV.

One positive study, conducted by Gray (2004), used aggregate national population data in 38 countries in sub-Saharan Africa to compare the percentage of Muslims in the population against the confirmed rate of HIV prevalence. He found a statistically significant negative association between HIV positivity and living in a Muslim country. Gray also examined six previous studies that link HIV, risk factors for HIV and Islamic faith to elucidate the exact nature of the protective relationship. Though a higher proportion of Muslims in a national population correlates with a lower HIV prevalence, confounding and contradictory factors raise doubts that religious affiliation alone explains this pattern. Gray credits the fact that Muslims are more likely to be circumcised with the majority of the protective effect since he found that Muslims did not appear to engage in fewer dangerous sexual behaviours than others in the same population. Gray's study may reveal an association, but does not credibly describe causation in the link between religious affiliation and HIV status.

Religion differs from many other cultural elements herein explored, in that it does not represent a discrete practice that imparts theoretical or practical risk. Without a clear link to a specific risk-enhancing behaviour, a focus on correlations between religion and HIV positivity only serves to inappropriately discredit certain religious groups and beliefs as culturally inferior. Blanket statements about religion, therefore, tend to essentialise groups perhaps even more egregiously than the more specific cultural practices reviewed above and do not offer any benefit in terms of identifying loci of intervention. This bias is doubly disturbing since the preponderance of evidence would suggest that religion and religiosity have little effect on the spread of HIV and, in keeping with other cultural phenomena, cannot account for the differences in global HIV prevalence.

## Conclusion

Culture and HIV/AIDS have been assumed to be inextricably linked since the disease was first described. Cultural assumptions about sub-Saharan Africans in particular were taken for granted early in the pandemic and shaped the search for sociocultural cofactors (Packard & Epstein 1991). Many of the proposed cultural risk factors were isolated from the ethnographic record and stripped of their cultural meanings, societal context and historical positioning, thereby decontextualising and reifying them (Gausset 2001; Packard & Epstein 1991; Schoepf 1995; Taylor 2007). The fixation on cultural causation has many negative consequences. An overemphasis on the role of cultural elements can easily be misdirected to blame afflicted societies for their own high rates of infection (AIDSnet & Bruun 2006). Particular cultural groups can also become stigmatised and ostracised by a fearful wider society on account of inappropriate claims of cultural causation (Parker & Aggleton 2003; Scambler 2009). The belief in cultural determinism conveniently releases the West of any responsibility for the plight of sub-Saharan Africa (AIDSnet & Bruun 2006). Moreover, it distracts stakeholders from developing interventions based on knowledge of true causative factors rather than assumptions that have in the end proved incorrect.

In recent years, as many of the proposed relationships between HIV/AIDS and culture have become controversial and tenuous, the trend in the literature has appropriately shifted towards a consideration of social structural factors as more important determinants of the disease (Parker 2001; Taylor 2007). The shift in focus conforms to calls by Packard and Epstein (1991:782) for illumination ‘by a more fundamental knowledge and understanding of the contours of African social and economic life, which involves more than a cataloging of risk behaviors’. These structural considerations include, amongst others, poverty, social inequality, migratory labour practices, food insecurity, inaccessibility to health care, unavailability of antiretroviral medications and political disempowerment. The role of structural factors is multifaceted and defies simplistic explanation, but generally fits the pattern that poorer, more marginalised groups are more likely to be affected by HIV/AIDS. The emphasis on social structure and power imbalances has the added benefit of recasting HIV prevention as an attempt not only to modify disease patterns, but also to mitigate social injustice and inequality (Parker 2001).

The trend towards de-emphasising culture as the driving force behind the prevalence of HIV/AIDS in sub-Saharan Africa is being supported by new ethnographic and epidemiological evidence, as herein presented. The proposed cultural risk factors in sub-Saharan Africa fail on inspection to explain global disparities in HIV prevalence because they are insufficiently dangerous, insufficiently widespread or insufficiently different from other parts of the world, where HIV/AIDS rates are far lower. Altogether, a careful consideration of the available evidence undermines attempts to blame an exotic cultural milieu for the high prevalence of HIV/AIDS. In short, support for culture as a singular determinant in the African epidemic of HIV/AIDS falls flat when disabused of its culturally biased and ethnocentric assumptions.
